# Comparison of the Predicted Population Coverage of Tuberculosis Vaccine Candidates Ag85B-ESAT-6, Ag85B-TB10.4, and Mtb72f via a Bioinformatics Approach

**DOI:** 10.1371/journal.pone.0040882

**Published:** 2012-07-17

**Authors:** Jose Davila, Lucy A. McNamara, Zhenhua Yang

**Affiliations:** 1 Department of Epidemiology, School of Public Health, University of Michigan, Ann Arbor, Michigan, United States of America; 2 Department of Microbiology and Immunology, University of Michigan, Ann Arbor, Michigan, United States of America; University of Texas at Tyler, United States of America

## Abstract

The Bacille-Calmette Guérin (BCG) vaccine does not provide consistent protection against adult pulmonary tuberculosis (TB) worldwide. As novel TB vaccine candidates advance in studies and clinical trials, it will be critically important to evaluate their global coverage by assessing the impact of host and pathogen variability on vaccine efficacy. In this study, we focus on the impact that host genetic variability may have on the protective effect of TB vaccine candidates Ag85B-ESAT-6, Ag85B-TB10.4, and Mtb72f. We use open-source epitope binding prediction programs to evaluate the binding of vaccine epitopes to Class I HLA (A, B, and C) and Class II HLA (DRB1) alleles. Our findings suggest that Mtb72f may be less consistently protective than either Ag85B-ESAT-6 or Ag85B-TB10.4 in populations with a high TB burden, while Ag85B-TB10.4 may provide the most consistent protection. The findings of this study highlight the utility of bioinformatics as a tool for evaluating vaccine candidates before the costly stages of clinical trials and informing the development of new vaccines with the broadest possible population coverage.

## Introduction

The Bacille-Calmette Guérin (BCG) vaccine is the single most widely administered vaccine in the world. More than half of the world’s population–over three billion people–had received the BCG vaccine by 2010 [1], [2]. Despite mass vaccination campaigns, however, tuberculosis (TB) has persisted as a serious public health problem in many areas [1]. This is in part because although BCG is effective against TB in early childhood, it offers only variable protection against adult pulmonary TB, the most infectious form of the disease [1]. As a result, it is estimated that one third of the world’s population is infected with *Mycobacterium tuberculosis*, and between two and three million people die from the disease every year [3].

Novel TB vaccines that aim to boost and/or replace BCG are currently in development, and some have shown promising results in *in vitro* studies, animal models, and phase I and II clinical trials [1], [2], [3], [4], [5], [6], [7], [8], [9], [10]. Success in these studies and trials may not accurately represent a vaccine’s protective coverage on the diverse global stage, however, as clinical trials are often limited in geographic area. Researchers have thus started to study the global coverage of novel vaccine candidates through interdisciplinary, pre-clinical approaches that integrate comparative genomics and bioinformatics in vaccine testing [11], [12], [13], [14]. Such integrated strategies have demonstrated great potential in their ability to harness readily accessible information on human and pathogen diversity to understand potential vaccine coverage.

A recent study from our laboratory sought to elucidate the joint impact of host and pathogen genetic variation on the predicted protective coverage of the polyprotein fusion TB vaccine candidate Mtb72f [13]. Building on previous work that found significant variations in the PPE18 protein of Mtb72f in a sample of clinical isolates [12], McNamara et al. performed *in silico* epitope binding predictions for Mtb72f epitopes and Class II Major Histocompatibility Complex (MHC) molecules, also known as Human Leukocyte Antigen (HLA) in humans. This study uncovered a set of Class II HLA alleles of high frequency in TB-endemic areas that were predicted to bind no or very few conserved Mtb72f epitopes. Given the importance of Class II HLA molecules in the human immune response to *M. tuberculosis* [3], the findings of this study point to high-TB burden populations where the protective effect of Mtb72f may be compromised by regional variation of Class II HLA alleles.

The present study employs *in silico* epitope binding predictions to assess and compare the predicted coverage of Ag85B-ESAT-6, Ag85B-TB10.4, and Mtb72f in populations with a high burden of TB. Expanding on our previous work [13], this study considered both Class I HLA-A, B, and C, and Class II HLA-DRB1 alleles. There are several reasons for examining Class II HLA-DRB1 diversity. Class II HLA proteins are responsible for stimulating CD4^+^ T cell-mediated destruction of phagocytosed pathogens, making Class II HLA especially important to the clearance of *M. tuberculosis* from macrophages [1]. Furthermore, proteins from the Class II HLA locus have been shown to have a predominant effect in the immunologic response to BCG [15]. Among Class II HLA genes, DR alleles bind the vast majority (90%) of the 500 known *M. tuberculosis* epitopes, and among DR alleles, DRB1 surface expression is five times greater than DRB3, DRB4, and DRB5 genes [16], [17]. Finally, epitope binding predictions for DRB1 alleles are more frequently available than other HLA Class II in prediction programs.

Although CD4+ T cell-mediated immunity is essential to combat *M. tuberculosis* infection, there is also evidence that CD8+ T cells are essential to the immune response to *M. tuberculosis* [18] and can recognize and eliminate *M. tuberculosis*-infected cells [19]. For this reason, we also investigated epitope binding to the major HLA Class I proteins, HLA–A, HLA–B, and HLA–C.

The Ag85B-ESAT-6 subunit vaccine candidate is composed of antigen 85B (Ag85B) and 6 kDa early secretory antigenic target (ESAT-6). Ag85B is a protein of the Ag85 complex that has been shown to be both highly conserved across mycobacterial species and highly immunogenic in animal models and humans [8], [9], [10], [20]. ESAT-6 is a virulence factor of low molecular mass that is restricted to bacteria of the TB complex and has been shown to be immunodominant among *M. tuberculosis* antigens [2]. This subunit vaccine demonstrated safety and immunogenicity in Phase I trials in human volunteers [21]. In addition, the H56-IC31 vaccine candidate developed by the Statens Serum Institut, Denmark, combines Ag85B and ESAT-6 with Rv2660 and IC31® adjuvant (Intercell). H56-IC31® is currently being tested for safety in a small group of healthy adults and adults with latent TB as part of Phase I clinical trials in South Africa [21].

Subunit vaccine candidate Ag85B-TB10.4 was created by the replacement of the ESAT-6 component of Ag85B-ESAT-6 with TB10.4. TB10.4 is a member of the ESAT-6 protein family and, like ESAT-6, is a low molecular mass, immunodominant protein [6]. The motivation behind exchanging ESAT-6 with TB10.4 is the high value of ESAT-6 as a diagnostic reagent and its previous use in commercially-available diagnostic tests [6]. Interestingly, TB10.4 has been shown to provoke a higher secretion of interferon gamma than ESAT-6 in TB patients [22]. H4-IC31®, a vaccine developed by SSI and Sanofi Pasteur (SP), combines Ag85B-TB10.4 (H4 antigen) with IC31® adjuvant in a BCG prime-boost regimen. H4-IC31® has completed Phase I clinical trials in Sweden, Finland, and South Africa, and is currently in a Phase I clinical trial in Switzerland [21], [23]. This vaccine will next be tested in Phase II infant efficacy trials and large Phase III adolescent and infant trials. Ag85B and TB10.4 have also been used in combination with Ag85A in an adenovirus vector (Ad35) BCG booster. This vaccine candidate, AERAS-402/Crucell Ad35, has completed three Phase I trials in the U.S. and is in ongoing Phase I and II clinical trials in South Africa, Kenya, and the U.S. [21].

Mtb72f, in contrast to the Ag85B vaccines, was found to have twenty-two populations of great concern and thirty-four populations of moderate concern for HLA–A alleles, one population of great concern and seven populations of moderate concern for HLA–B alleles, twenty-eight populations of moderate concern for HLA–C alleles, and two populations of great concern and one population of moderate concern for HLA-DRB1 alleles (Tables 3, 4, 5, 6, 7). In total, it is predicted that 30% or more of the population in twenty-five populations from high TB burden countries will be homozygous for HLA molecules that bind four or fewer Mtb72f vaccine epitopes for at least one HLA locus, and ninety-five populations from high TB burden countries are estimated to have a population frequency of 10% or greater of individuals homozygous for HLA molecules that are predicted to bind four or fewer vaccine epitopes for at least one HLA locus.

The Mtb72f subunit vaccine is composed of the two proteins PPE18, a member of the PPE protein family with an as yet unknown function, and pepA, a putative serine protease [12]. GSK M72, a vaccine candidate containing Mtb72f, is in ongoing Phase II clinical trials in a small cohort of infants in The Gambia and has completed Phase I clinical trials in Belgium and Phase II clinical trials in South Africa. GSK M72 was developed by GlaxoSmithKline as a BCG prime-boost candidate, and will next undergo testing in a cohort of 45 healthy, BCG-vaccinated adults in South Africa [21].

Ag85B-ESAT-6, Ag85B-TB10.4, and Mtb72f have all shown the potential to induce protective immunity against TB infection. The aims of this study are twofold. First, we hope to model a novel, cost-effective, and open-access method for the assessment of promising TB vaccine candidates as they progress into the costly stages of clinical trials. Second, we wish to provide additional insight into the predicted coverage of these three TB vaccine candidates in a manner that may inform the selection of test populations for future clinical trials.

## Results

### MHC Class I binding Predictions for Ag85B-ESAT-6, Ag85B-TB10.4, and Mtb72f

Binding predictions for Ag85B-TB10.4, Ag85B-ESAT-6, and Mtb72f were generated for 89 Class I HLA alleles representing the three most common alleles of each of the three Class I genes – HLA–A, HLA–B, and HLA–C – in populations with a high burden of TB identified by the World Health Organization (WHO) [24]. Class I allele frequencies in these populations were determined using the online database Allele*Frequencies in Worldwide Populations [25]. Epitope binding predictions were generated with NetMHCcons, a consensus method server that integrates artificial neural network (ANN), pan-specific ANN, and matrix-based methods for high-accuracy predictions [26]. NetMHCcons was recently determined to be the best available method for generating MHC Class I predictions [27].

Epitope binding predictions were generated for conserved vaccine epitopes. Recent studies from our lab reported that sixty percent of PPE18 epitopes and all pepA, Ag85B, ESAT-6, and TB10.4 epitopes are conserved [11], [13]. The number of vaccine epitopes predicted to bind any one MHC I allele ranged from 1 to 43 for Ag85B-ESAT-6, from 1 to 52 for Ag85B-TB10.4, and from 0 to 43 for Mtb72f. Only minor differences were observed in the number of predicted bindings between Ag85B-ESAT-6 and Ag85B-TB10.4, while greater discrepancies were observed between Mtb72f and the Ag85B vaccines. Four Class I HLA alleles were predicted to bind zero conserved Mtb72f epitopes (HLA–A*3301, A*7401, B*4002, and B*4006), and 36 of 89 (40%) Class I HLA alleles were predicted to bind four or fewer conserved Mtb72f epitopes–a designation termed “allele of concern” by McNamara et al. [13]. In contrast, all Class I HLA alleles were predicted to bind at least one epitope of Ag85B-ESAT-6 and Ag85B-TB10.4. Ag85B-ESAT-6 was found to have 14 (16%) alleles of concern while Ag85B-TB10.4 was found to have 11 (12%) (Tables S1, S2, S3).

### MHC Class I Alleles of Greatest Concern

Four Class I HLA alleles were predicted to bind no Mtb72f epitopes: HLA- A*3301, A*7401, B*4002, and B*4006. These alleles are among the three most prevalent alleles in the following populations: Bangladesh Dhakha Bangalee; China Harbin Korean and Inner Mongolian; India Andhra Pradesh Golla, Delhi, Kerala, Khandesh Region Parwa, Mumbai Maratha, North, West Bhil; Kenya; Pakistan Karachi Parsi; Russia Bering Island Aleut and Tuva; South Africa Natal Tamil; Uganda Kampala (Tables S1, S2). All of these populations belong to one of the 22 high TB burden countries identified by the WHO [24].

### MHC Class I Supertype Alleles

**Figure 1:**
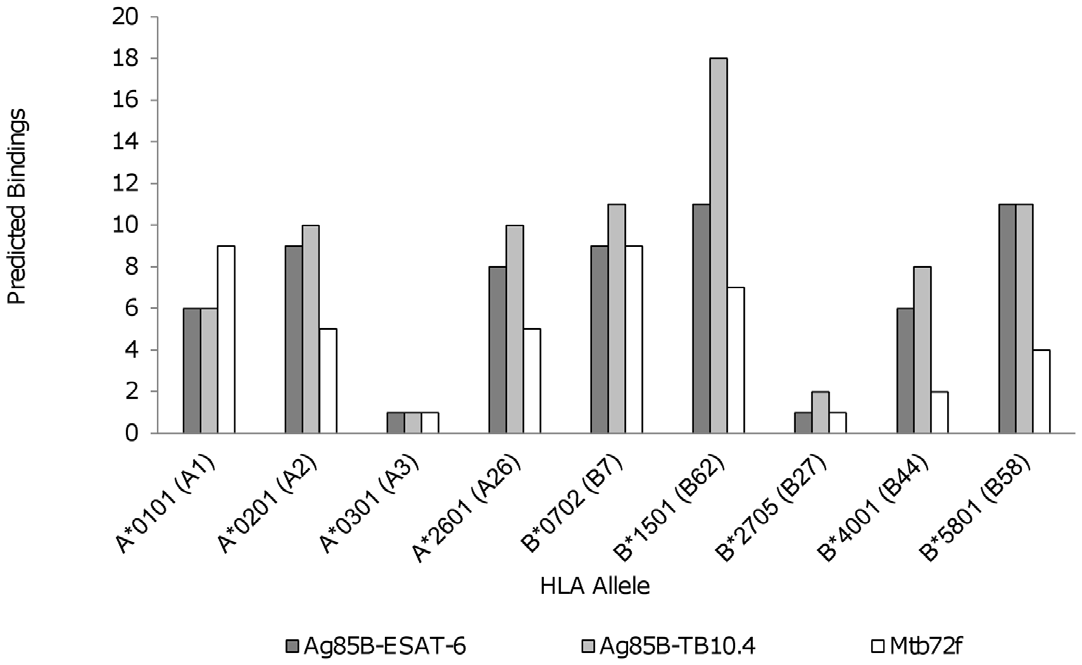
Supertype Class I HLA-, -B, and –C alleles. A comparison of the number of Ag85B-ESAT-6, Ag85B-TB10.4, and Mtb72f vaccine epitopes predicted to bind to each of the nine Class I HLA supertype alleles.

Nine HLA Class I supertypes, or supermotifs with binding properties similar to a large number of Class I HLA allelic variants, were used to compare the predicted bindings of Ag85B-ESAT-6, Ag85B-TB10.4, and Mtb72f (Figure 1). These alleles were: HLA- A*0101, A*0201, A*0301, A*2601, B*0702, B*1501, B*2705, B*4001, and B*5801 [28]. Ag85B-TB10.4 had the highest number of epitopes predicted to bind to supertype alleles for six of the nine supertypes: A*0201, A*2601, B*0702, B*1501, B*2705, and B*4001. Ag85B-TB10.4 and Ag85B-ESAT-6 had the same number of epitopes predicted to bind B*5801, and all three vaccines had the same number of epitopes predicted to bind to A*0301. Finally, Mtb72f had a higher number of predicted bindings than either Ag85B vaccine for just one supertype: A*0101. Three of the nine supertype alleles – A*0301, B*2705, and B*4001– were alleles of concern for Mtb72f, while only A*0301 and B*2705 were alleles of concern for Ag85B-ESAT-6 and Ag85B-TB10.4.

### MHC Class II Binding Predictions for Ag85B-ESAT-6, Ag85B-TB10.4, and Mtb72f

Binding predictions for Ag85B-ESAT-6, Ag85B-TB10.4, and Mtb72f were generated for 34 HLA-DRB1 alleles representing the three most common DRB1 alleles in each of the populations in the Allele*Frequencies in Worldwide Populations databank from the 22 countries with the highest burden of TB as identified by the WHO [24], [25]. Epitope binding predictions were generated with ARB, NetMHCII, NetMHCIIpan, ProPred, SVRMHCII, MHCPred, RankPEP, and Vaxign [13]. Like NetMHCcons, the selection of programs for Class II predictions took a consensus-method approach that included ANN, support vector machine regression, matrix-based, and partial least squares methods. Wherever possible, multiple epitope prediction programs were used to generate a median number of binding predictions for each allele. The median number of vaccine epitopes predicted to bind any one DRB1 allele ranged from 3 to 83 for Ag85B-ESAT-6, from 5 to 82 for Ag85B-TB10.4, and from 0 to 79 for Mtb72f (Table S4).

Epitope binding performance followed a trend similar to the one observed in the Class I HLA binding predictions. Minor differences in the number of epitopes predicted to bind each allele were observed between Ag85B-ESAT-6 and Ag85B-TB10.4, while greater discrepancies emerged between Mtb72f and the Ag85B vaccines. Mtb72f was found to have seven alleles of concern (DRB1*0302, *0403, *0411, *0807, *1401, *1403, and *1502). Ag85B-ESAT-6 was found to have two alleles of concern (DRB1*0801 and *0807), while Ag85B-TB10.4 had no alleles of concern.

### MHC Class II Alleles of Greatest Concern

Two Class II HLA-DRB1 alleles were predicted to bind no Mtb72f epitopes: *0302 and *1403. These alleles are among the three most prevalent in the Venda population of South Africa, China Yunnan Province’s Drung, and the Evenki and Ket populations of Russia (Table S4). All of these populations belong to one of the 22 high TB burden countries identified by the WHO [24].

### MHC Class II Supertype Alleles

**Figure 2:**
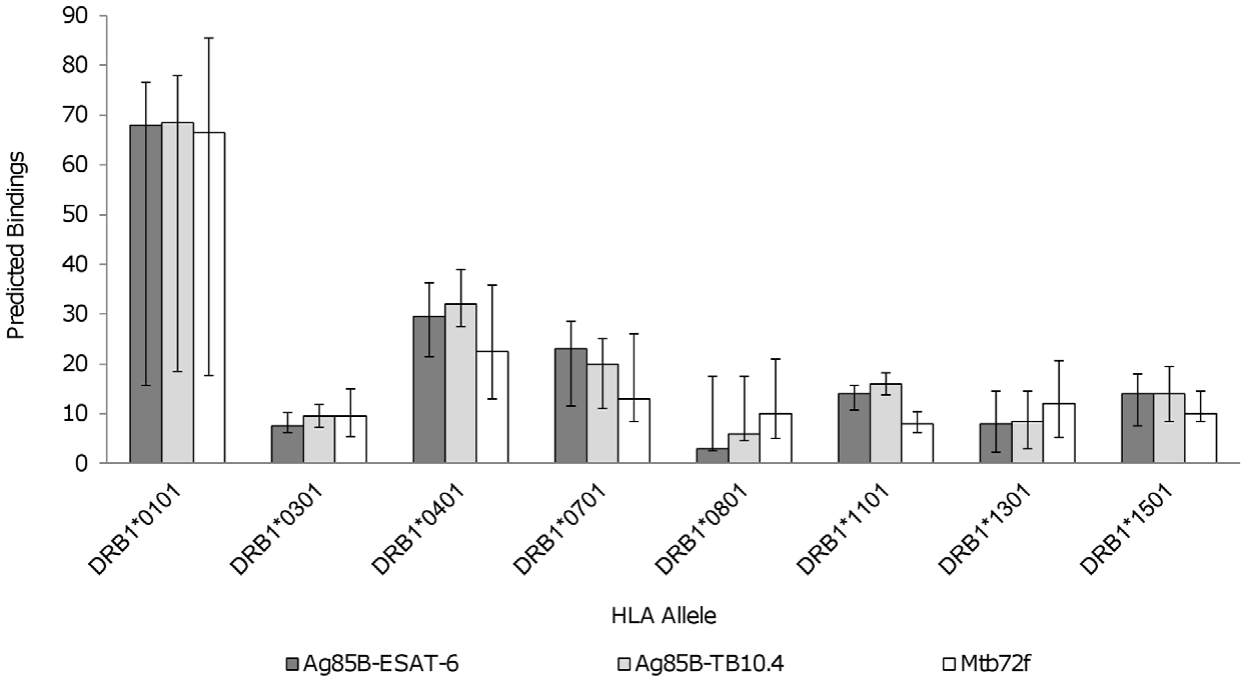
Supertype Class II HLA-DRB1 alleles. A comparison of the median number of Ag85B-ESAT-6, Ag85B-TB10.4, and Mtb72f vaccine epitopes predicted to bind to each of the eight HLA-DRB1 supertype alleles. Median and interquartile ranges of the epitopes predicted to bind by each of the eight prediction programs used are shown.

Eight Class II HLA supertype alleles, or supermotifs with binding properties similar to a large number of Class II HLA allelic variants, were used to compare binding predictions among Ag85B-ESAT6, Ag85B-TB10.4, and Mtb72f. The eight supertype alleles were: DRB1*0101, *0301, *0401, *0701, *0801, *1101, *1301, and *1501 [29]. Mtb72f was predicted to have fewer binding epitopes than either Ag85B-ESAT6 or Ag85B-TB10.4 for five of the eight supertype alleles (DRB1*0101, DRB1*0401, DRB1*0701, DRB1*1101, and DRB1*1501) (Figure 2). However, Mtb72f had more epitopes than the Ag85B vaccines that were predicted to bind to DRB1*0801 and DRB1*1301. Ag85B-TB10.4 was predicted to have more binding epitopes than Ag85B-ESAT-6 or Mtb72f for three of the eight alleles (DRB1*0101, DRB1*0401, and DRB1*1101) (Figure 2). Ag85B-ESAT-6 had the most epitopes predicted to bind to DRB1*0701. Both Ag85B vaccines had the same number of epitopes predicted to bind to DRB1*1501, while Mtb72f had fewer epitopes predicted to bind this allele. Finally, Mtb72f and Ag85B-TB10.4 had the same number of epitopes predicted to bind to DRB1*0301 while Ag85B-ESAT-6 had fewer. Only Ag84B-ESAT-6 was found to have a supertype allele of concern, HLA*0801, which is found at high frequency in the Ket population of Russia.

### Populations of Concern

Allele frequencies of MHC Class I and II alleles of concern were considered to assess the population coverage of the three vaccine candidates, and all populations were classified as being of lesser, moderate, or great concern. Populations of moderate concern were defined as populations where the frequency of individuals with two HLA alleles of the same HLA gene that are both alleles of concern–alleles predicted to bind four or fewer vaccine epitopes–was 10% or greater and less than 30%. Populations of great concern were defined as those where the frequency of having both HLA alleles be alleles of concern was 30% or greater. All other populations were classified as being of lesser concern. The frequency of individuals with two alleles of concern was calculated using the assumption that allele frequencies in the population adhere to Hardy-Weinberg equilibrium allele frequencies.

Vaccine candidate Ag85B-ESAT-6 was found to have five populations of great concern and seventeen populations of moderate concern for HLA-A alleles, no populations of concern for HLA-B or HLA-C alleles, and no populations of concern in our analysis of HLA-DRB1 alleles, (Table 1). The five populations of great concern for Ag84B-ESAT-6 were the Chinese Wa, Hani, Dai, and Jinuo popuations, and Indian Puyala population.

Ag85B-TB10.4 was similarly found to have no populations of concern for HLA-DRB1 alleles, HLA-B alleles, and HLA–C alleles. We found three populations of great concern and nine populations of moderate concern in our analysis of HLA–A allele frequencies (Table 2). The three populations of great concern were the Chinese Wa and Hani populations and the Indian Puyala population.

**Table 1:** Populations of moderate and great concern for Ag85B-ESAT-6.

Gene	Population of concern	Allele of concern 1	f*	Allele of concern 2	f*	Allele of concern 3	f*	Phenotype Frequency
HLA–A	China Guangxi Region Maonan	A*1101	0.352	A*0207	0.134			0.236
	China Guizhou Province Bouyei	A*1101	0.314	A*0207	0.227			0.293
	China Guizhou Province Miao pop 2	A*1101	0.359	A*0207	0.165			0.275
	China Guizhou Province Shui	A*1101	0.295	A*0207	0.175			0.221
	China South Han	A*1101	0.277	A*3303	0.115			0.154
	China Southwest Dai**	A*1101	0.391	A*0207	0.185			0.332
	China Yunnan Province Bulang	A*1101	0.543					0.295
	China Yunnan Province Han	A*1101	0.317	A*0207	0.183			0.250
	China Yunnan Province Hani pop 2**	A*1101	0.613	A*0207	0.107			0.518
	China Yunnan Province Jinuo**	A*1101	0.367	A*0207	0.188			0.308
	China Yunnan Province Wa**	A*1101	0.584	A*3303	0.160			0.554
	India Kerala Hindu Pulaya**	A*1101	0.531	A*0301	0.063			0.353
	India New Delhi	A*1101	0.235	A*0301	0.098			0.111
	India Tamil Nadu Nadar	A*0301	0.205	A*3101	0.189			0.155
	Indonesia Java Western	A*1101	0.164	A*3303	0.162			0.106
	Indonesia Sudanese and Javanese	A*3303	0.169	A*1101	0.164			0.111
	Pakistan Baloch	A*1101	0.222	A*3303	0.127			0.122
	Pakistan Burusho	A*3303	0.179	A*0301	0.130	A*1101	0.125	0.188
	Thailand	A*1101	0.299	A*0207	0.109			0.167
	Thailand Northeast	A*1101	0.271	A*0207	0.157			0.183
	Thailand Northeast pop 2	A*1101	0.233	A*0207	0.144			0.142
	Vietnam Hanoi Kinh pop 2	A*1101	0.229	A*3303	0.115			0.118
HLA–B	None	None						–
HLA–C	None	None						–
HLA-DRB1	None	None						–

* Allele frequency, from the Allele*frequencies database.

** Populations of great concern, defined as populations where 30% or more of the population has an expected phenotype of reduced protection by the vaccine due to having two alleles of concern for a single HLA locus, assuming Hardy-Weinberg equilibrium. Alleles of concern are defined as alleles predicted to bind four or fewer vaccine epitopes.

**Table 2:** Populations of moderate and great concern for Ag85B-TB10.4.

Gene	Population of concern	Allele ofconcern 1	f*	Allele ofconcern 2	f*	Allele ofconcern 3	f*	Phenotype Frequency
HLA–A	Bangladesh Dhaka Bangalee	A*3301	0.170	A*1101	0.156			0.106
	China Yunnan Province Bulang	A*1101	0.543					0.295
	China Yunnan Province Hani pop 2**	A*1101	0.613					0.376
	China Yunnan Province Wa**	A*1101	0.584	A*3303	0.160			0.554
	India Kerala Hindu Pulaya**	A*1101	0.531	A*0301	0.063	A*3301	0.063	0.432
	India New Delhi	A*1101	0.235	A*0301	0.098			0.111
	India Tamil Nadu Nadar	A*0301	0.205	A*3101	0.189			0.155
	Indonesia Java Western	A*1101	0.164	A*3303	0.162			0.106
	Indonesia Sudanese and Javanese	A*3303	0.169	A*1101	0.164			0.111
	Pakistan Baloch	A*1101	0.222	A*3303	0.127			0.122
	Pakistan Brahui	A*1101	0.252	A*3201	0.092			0.118
	Pakistan Burusho	A*3303	0.179	A*0301	0.130	A*1101	0.125	0.188
HLA–B	None	None						–
HLA–C	None	None						–
HLA-DRB1	None	None						–

* Allele frequency, from the Allele*frequencies database.

** Populations of great concern, defined as populations where 30% or more of the population has an expected phenotype of reduced protection by the vaccine due to having two alleles of concern for a single HLA locus, assuming Hardy-Weinberg equilibrium. Alleles of concern are defined as alleles predicted to bind four or fewer vaccine epitopes.

**Table 3:** Populations of moderate and great concern for Mtb72f based on HLA–A allele.

Population of concern	Allele of concern 1	f*	Allele of concern 2	f*	Allele of concern 3	f*	Phenotype Frequency
Bangladesh Dhaka Bangalee	A*2402	0.163	A*1101	0.156			0.102
Brazil Parana Oriental	A*2402	0.227	A*1101	0.121			0.121
Brazil Terena	A*6801	0.250	A*3101	0.183			0.187
China Canton Han	A*1101	0.267	A*2402	0.163			0.185
China Guangdong Province	A*1101	0.303	A*2402	0.137			0.194
China Guangdong Province Meizhou Han**	A*1101	0.303	A*2402	0.222	A*2420	0.116	0.411
China Guangxi Region Maonan**	A*1101	0.352	A*0207	0.134	A*2402	0.134	0.384
China Guizhou Province Bouyei**	A*1101	0.314	A*0207	0.227	A*2402	0.139	0.462
China Guizhou Province Miao pop 2**	A*1101	0.359	A*0207	0.165	A*2402	0.147	0.450
China Guizhou Province Shui**	A*1101	0.295	A*2402	0.243	A*0207	0.175	0.508
China Harbin Manchu	A*2402	0.166	A*1101	0.162			0.108
China Inner Mongolian Region	A*2402	0.196	A*1101	0.162			0.128
China Qinghai Province Hui	A*2402	0.164	A*1101	0.159			0.104
China Shaanxi Province Han	A*1101	0.187	A*2402	0.158			0.119
China Shandong Province Linqu County	A*1101	0.204	A*3002	0.186			0.152
China Shanghai	A*1101	0.226	A*2402	0.173			0.159
China South Han**	A*1101	0.277	A*2402	0.172	A*3303	0.115	0.318
China Southwest Dai**	A*1101	0.391	A*0207	0.185			0.332
China Tibet Region Tibetan	A*2402	0.272	A*1101	0.130			0.162
China Wuhan	A*1101	0.293	A*2402	0.178			0.222
China Yunnan Province Bulang**	A*1101	0.543	A*2402	0.237	A*2407	0.103	0.780
China Yunnan Province Han pop 2	A*2402	0.316	A*1101	0.123			0.193
China Yunnan Province Han**	A*1101	0.317	A*0207	0.183	A*2402	0.163	0.440
China Yunnan Province Hani pop 2**	A*1101	0.613	A*0207	0.107	A*2402	0.090	0.656
China Yunnan Province Jinuo**	A*1101	0.367	A*0207	0.188	A*2402	0.183	0.545
China Yunnan Province Lisu**	A*1101	0.455	A*2402	0.118			0.328
China Yunnan Province Naxi**	A*1101	0.380	A*2402	0.176			0.309
China Yunnan Province Nu**	A*1101	0.481	A*2402	0.114			0.354

* Allele frequency, from the Allele*frequencies database.

** Populations of great concern, defined as populations where 30% or more of the population has an expected phenotype of reduced protection by the vaccine due to having two alleles of concern for a single HLA locus, assuming Hardy-Weinberg equilibrium. Alleles of concern are defined as alleles predicted to bind four or fewer vaccine epitopes.

**Table 4:** Populations of moderate and great concern for Mtb72f based on HLA–A allele (continued).

Population of concern	Allele of concern 1	f*	Allele of concern 2	f*	Allele of concern 3	f*	Allele of concern 4	f*	Phenotype Frequency
China Yunnan Province Wa**	A*1101	0.584	A*3303	0.160	A*2402	0.130			0.764
India Kerala Hindu Nair	A*2402	0.232	A*0301	0.146					0.143
India Kerala Hindu Pulaya**	A*1101	0.531	A*2402	0.250	A*0301	0.063			0.712
India Khandesh Region Pawra	A*1101	0.210	A*2402	0.160					0.137
India Mumbai Maratha	A*2402	0.167	A*3303	0.130	A*1101	0.123			0.176
India New Delhi	A*1101	0.235	A*2402	0.114	A*0301	0.098			0.200
India North pop 2	A*2402	0.192	A*1101	0.125					0.100
India North pop 3	A*2402	0.198	A*1101	0.172					0.137
India Tamil Nadu Nadar**	A*0301	0.205	A*3101	0.189	A*2402	0.156			0.303
India West Bhil	A*3303	0.180	A*2407	0.150					0.109
Indonesia Java pop 2	A*2407	0.264	A*1101	0.139	A*2402	0.139			0.294
Indonesia Java Western**	A*2407	0.222	A*1101	0.164	A*3303	0.162			0.300
Indonesia Sudanese and Javanese	A*2407	0.207	A*3303	0.169	A*1101	0.164			0.292
Pakistan Baloch	A*1101	0.222	A*3303	0.127					0.122
Pakistan Burusho**	A*3303	0.179	A*0301	0.130	A*1101	0.125	A*2402	0.125	0.312
Russia Arkhangelsk Pomor	A*0301	0.160	A*2402	0.160					0.102
Russia Bering Island Aleut	A*2402	0.241	A*0301	0.129					0.137
Russia Chuvash	A*2402	0.189	A*0301	0.158					0.120
Russia Murmansk Saomi Mixed	A*2402	0.260	A*0301	0.180					0.194
Russia Nenet Mixed	A*2402	0.375	A*0301	0.172					0.299
Russia Sakhalin island Nivkhi**	A*2402	0.509	A*3002	0.057					0.320
South Africa Natal Tamil	A*1101	0.180	A*2402	0.160					0.116
Thailand	A*1101	0.299	A*0207	0.109					0.166
Thailand Northeast pop 2**	A*1101	0.233	A*2402	0.188	A*0207	0.144			0.319
Thailand Northeast**	A*1101	0.271	A*2402	0.196	A*0207	0.157			0.389
Thailand pop 4	A*1101	0.277	A*2402	0.173					0.203
Vietnam Hanoi	A*1101	0.330	A*2402	0.130					0.212
Vietnam Hanoi Kinh pop 2	A*1101	0.229	A*2402	0.138	A*3303	0.115			0.232

* Allele frequency, from the Allele*frequencies database.

** Populations of great concern, defined as populations where 30% or more of the population has an expected phenotype of reduced protection by the vaccine due to having two alleles of concern for a single HLA locus, assuming Hardy-Weinberg equilibrium. Alleles of concern are defined as alleles predicted to bind four or fewer vaccine epitopes.

**Table 5:** Populations of moderate and great concern for Mtb72f based on HLA–B allele.

Population of concern	Allele of concern 1	f*	Allele of concern 2	f*	Allele of concern 3	f*	Phenotype Frequency
China Guangdong Province Meizhou Han	B*5801	0.170	B*4001	0.155			0.106
China Guangxi Region Maonan	B*1301	0.199	B*4001	0.134			0.111
China Shandong Province Linqu County	B*1301	0.220	B*4001	0.124			0.118
India Kerala Kuruma	B*5802	0.333	B*4001	0.200			0.284
India Kerala Malapandaram**	B*4001	0.450	B*5801	0.250	B*5701	0.200	0.810
India Khandesh Region Pawra	B*4006	0.170	B*5801	0.150			0.102
Russia Sakhalin island Nivkhi	B*4001	0.312	B*4801	0.113	B*2704	0.104	0.280
South Africa Tswana	B*5802	0.220	B*4403	0.111			0.110

* Allele frequency, from the Allele*frequencies database.

** Populations of great concern, defined as populations where 30% or more of the population has an expected phenotype of reduced protection by the vaccine due to having two alleles of concern for a single HLA locus, assuming Hardy-Weinberg equilibrium. Alleles of concern are defined as alleles predicted to bind four or fewer vaccine epitopes.

**Table 6:** Populations of moderate and great concern for Mtb72f based on HLA–C allele.

Population of concern	Allele of concern 1	f*	Allele of concern 2	f*	Allele of concern 3	f*	Phenotype Frequency
Brazil Pernambuco Mixed	C*0401	0.228	C*0602	0.109			0.114
Brazil Terena	C*0401	0.223	C*0702	0.202			0.181
China Guangdong Province Meizhou Han	C*0702	0.258	C*0717	0.147			0.164
China Yunnan Province Bulang	C*0702	0.190	C*0401	0.134			0.105
China Yunnan Province Lisu	C*0702	0.329					0.108
China Yunnan Province Nu	C*0702	0.307	C*0401	0.157			0.215
India Delhi pop 2	C*0602	0.136	C*0401	0.117	C*0702	0.099	0.124
India Kerala Hindu Ezhava	C*0702	0.229	C*0401	0.146			0.141
India Kerala Hindu Namboothiri	C*0401	0.213	C*0702	0.213			0.181
India Kerala Hindu Pulaya	C*0401	0.188	C*0702	0.188			0.141
India Kerala Kattunaikka	C*0401	0.412					0.170
India Kerala Kurichiya	C*0702	0.350	C*0401	0.150			0.250
India Kerala Malabar Muslim	C*0401	0.279	C*0702	0.147			0.181
India Kerala Syria Christian	C*0401	0.226	C*0702	0.145			0.138
India Mumbai Maratha	C*0602	0.222	C*0401	0.154	C*0704	0.099	0.226
India North pop 2	C*0401	0.265	C*0702	0.162			0.182
India Tamil Nadu Nadar	C*0401	0.213	C*0702	0.148			0.130
India West Coast Parsi	C*0602	0.240	C*0401	0.110			0.123
Kenya Luo	C*0602	0.187	C*0401	0.132			0.102
Kenya Nandi	C*0602	0.217	C*0401	0.115			0.110
Pakistan Burusho	C*0702	0.255	C*0401	0.133			0.151
Pakistan Karachi Parsi	C*0602	0.214	C*0401	0.181			0.156
Pakistan Mixed Pathan	C*0401	0.165	C*0702	0.160	C*0602	0.120	0.198
Russia Arkhangelsk Pomor	C*0702	0.260	C*0602	0.130	C*0401	0.120	0.260
Russia Moscow	C*0702	0.257	C*0602	0.130			0.150
Russia Murmansk Saomi Mixed	C*0401	0.190	C*0702	0.140			0.109
Thailand Northeast	C*0702	0.271	C*0401	0.131			0.162
Uganda Kampala pop 2	C*0602	0.191	C*0401	0.160			0.123

* Allele frequency, from the Allele*frequencies database.

** Populations of great concern, defined as populations where 30% or more of the population has an expected phenotype of reduced protection by the vaccine due to having two alleles of concern for a single HLA locus, assuming Hardy-Weinberg equilibrium. Alleles of concern are defined as alleles predicted to bind four or fewer vaccine epitopes.

**Table 7:** Populations of moderate and great concern for Mtb72f based on HLA-DRB1 allele.

Population of concern	Allele of concern 1	f*	Allele of concern 2	f*	Phenotype Frequency
China Yunnan Province Drung**	DRB1*1401	0.807	DRB1*1403	0.043	0.723
Brazil East Amazon**	DRB1*0411	0.630			0.397
Brazil Ticuna	DRB1*0411	0.316	DRB1*0807	0.224	0.292

* Allele frequency, from the Allele*frequencies database.

** Populations of great concern, defined as populations where 30% or more of the population has an expected phenotype of reduced protection by the vaccine due to having two alleles of concern for a single HLA locus, assuming Hardy-Weinberg equilibrium. Alleles of concern are defined as alleles predicted to bind four or fewer vaccine epitopes.

### Testing Epitope Predictions with Control Proteins

In order to test whether observed variations in predicted epitope bindings were a function of the vaccine proteins and not an artifact of the prediction programs, we analyzed MHC Class I and Class II epitope binding predictions for three non-mycobacterium control proteins in addition to the vaccine proteins (Tables S1, S2, S3, S4). The control proteins used were of similar amino acid length to the vaccine candidates and included: 1) Dihydrolipoyllysine-residue succinyltransferase (389 aa) of *Neisseria meningitides,* 2) Cytochrome B (380 aa) of *Homo sapiens*, and 3) TPA_exp: BimA (373 aa) of *Burkholdereria mallei* (www.ncbi.nlm.nih.gov). We then performed a 2-way ANOVA on control and test protein epitope predictions for all Class I and Class II alleles analyzed. We found that for Class I epitope prediction data, different HLA–A, −B, and −C alleles account for 51.59% of the variation in epitopes predicted to bind (F = 10.55, p<0.0001) while the specific vaccine or control protein analyzed accounts for 23.52% of the variation (F = 84.16, p<0.0001). For the Class II predictions, different HLA-DRB1 alleles account for 66.55% of the variation in epitopes predicted to bind (F = 37.29, p<0.0001) while the specific vaccine or control protein analyzed accounts for 24.52% of the variation (F = 90.70, p<0.0001). Although the vaccine and control proteins follow generally the same pattern as far as the alleles to which relatively few or many epitopes are predicted to bind, these findings demonstrate that the number of epitopes predicted to bind each DRB1 allele varies significantly by the choice of protein or vaccine analyzed.

## Discussion

The potential impact of microbial and host genetic diversity on the protective coverage of novel TB vaccines has not been assessed until recently [11], [12], [13]. To explore the potential impact of host genetic diversity on the population coverage of three TB vaccine candidates, Ag85B-ESAT-6, Ag85B-TB10.4, and Mtb72f, we conducted epitope binding predictions of vaccine epitopes to Class I and Class II HLA alleles. Epitope binding predictions for these vaccine candidates were compared to assess the relative predicted coverage of the three vaccines.

We defined HLA alleles of concern for a given vaccine as alleles predicted to bind 4 or fewer vaccine epitopes. Among HLA Class I allelic variants of high frequency in TB endemic regions, a much higher number (37) of alleles of concern was found for Mtb72f than for the Ag85B vaccines (11 for Ag85B-TB10.4 and 14 for Ag85B-ESAT6). There were fewer Class II HLA-DRB1 alleles of concern, but a similar trend in the number of alleles of concern for each vaccine candidate was observed. Binding predictions generated the greatest number (7) of alleles of concern for Mtb72f and fewer alleles of concern (2 and 0, respectively) for Ag85B-ESAT-6 and Ag85B-TB10.4. Furthermore, four Class I alleles and two Class II alleles were predicted to bind no Mtb72f epitopes, termed “alleles of greatest concern” for this vaccine candidate.

We also defined populations of moderate and great concern for each vaccine as those in which a substantial proportion of the population would have two alleles of concern for a single HLA locus. Populations of moderate concern were defined as those where between 10% and 30% of the population has two alleles of concern at a given HLA locus; populations of great concern were defined as those where 30% of the population fulfills this criterion. Mtb72f was found to have the greatest numbers of populations of moderate and great concern among the three vaccine candidates, with three populations of concern based on HLA-DRB1 alleles, 56 based on HLA-A, 8 based on HLA–B, and 28 based on HLA–C. Ag85B-ESAT-6 and Ag85B-TB10.4 were each found to have no populations of concern based on HLA-DRB1, HLA-B, and HLA-C alleles, and were found to have 22 and 12 populations of moderate or great concern, respectively, based on HLA-A alleles.

Ag85B-TB10.4 generally had more predicted epitope bindings per allele than Ag85B-ESAT-6. Ag85B-TB10.4 also had the fewest alleles of concern and the fewest populations of concern, as defined above. The observed difference between Ag85B-ESAT-6 and Ag85B-TB10.4 has an important implication in the development of new TB vaccines because ESAT-6 is a key component in a new generation of vaccine candidates against *M. tuberculosis* infection [4], [30]. One particularly promising vaccine candidate is H56-IC31®, which includes the component proteins Ag85B, ESAT-6, and Rv2660c [4]. Given the findings of this study, the TB10.4 protein may be considered as an alternative to include in a multistage TB vaccine, as it may confer more consistent protection in the global population. ESAT-6 has also been reported as an important component in *M. tuberculosis* diagnostics; Ag85B-TB10.4 was in fact developed as a sequel to Ag85B-ESAT-6 to maintain the viability of ESAT-6-based immunological assays in immunized individuals [6]. The finding of this study that Ag85B-TB10.4 may provide broader and more consistent coverage than Ag85B-ESAT-6 and Mtb72f provides additional incentive to use TB10.4 instead of the ESAT-6 subunit.

It is essential to note that, of the epitopes predicted to bind an HLA molecule, not all will actually be bound by these alleles in vivo. Before being bound by class I and class II HLA molecules, epitopes must undergo processing and, because not all possible epitopes will actually be generated through intracellular processing, not all epitopes predicted to bind may be present *in vivo* to activate a protective immune response. As there currently exists no accurate means of determining which epitopes will be generated *in vivo*, *in silico* epitope binding predictions are overestimates of *in vivo* epitope bindings. This fact suggests that *in silico* alleles of concern may be of even more serious concern *in vivo*, binding fewer epitopes than predicted or none at all. Furthermore, even if an epitope is presented on an HLA molecule, the specific epitope/HLA molecule combination may not be strongly immunogenic. The distal impact of these points is that a vaccine candidate may not succeed in inducing immunity in individuals with HLA genotypes predicted to bind very few of the vaccine’s epitopes: few or none of the epitopes predicted to bind may actually be generated in vivo, and if they are generated they still may not stimulate a strong immune response.

The ranges of Ag85B-ESAT-6, Ag85B-TB10.4, and Mtb72f epitopes predicted to bind allelic variants of Class I and II demonstrate considerable variation: 0 to 52 epitopes predicted to bind among Class I alleles and 0 to 83 among Class II alleles. As evinced by the distribution of the number of predicted bindings (Tables S1, S2, S3, S4), some Class I or II alleles are predicted to bind a high number of epitopes from all three vaccines, whereas others are predicted to bind relatively few epitopes from all three vaccines. This is consistent with our finding that the majority of the variation in the number of epitopes from the various vaccines and control proteins predicted to bind each HLA molecule can be accounted for by differences among DRB1 or Class I alleles. This finding is not surprising because different HLA alleles recognize different amino acid patterns within epitopes, and some alleles have less stringent recognition criteria (i.e. more amino acids permitted at specific locations within the epitope core) and/or recognize epitopes containing more common amino acids. Because of these differences in recognition criteria, substantial differences in the frequency of epitopes that are able to bind to each HLA allele are expected. We furthermore found that the number of epitopes predicted to bind each allele also varies significantly when different test and control proteins are used to generate predictions. This analysis agrees with our overall epitope prediction results, which suggest that the level of protection conferred by any one vaccine candidate will vary among people with different genetic backgrounds, and also that a single vaccine candidate will not be more effective than the others in people of every genotype.

As demonstrated by McNamara et al. [13], pathogen diversity can have a substantial impact on the outcomes of epitope binding predictions. In particular, genetic diversity may introduce or remove epitopes that are important to the vaccine’s interaction with Class I and Class II HLA molecules. In the current study, we focused on the diversity of human Class I and Class II HLA alleles rather than the genetic diversity of Ag85B-ESAT-6 and Ag85B-TB10.4, because a previous study from our laboratory found no sequence variation in the *M. tuberculosis* genes encoding the protein components of Ag85B-ESAT-6 and Ag85B-TB10.4 among 101 *M. tuberculosis* clinical strains from Arkansas and Turkey [11]. However, a recent study found that TB10.4 may actually have more diversity than most other TB genes [31], which would complicate the predicted interactions between HLA molecules and vaccine epitopes. Additional studies using samples representing different genetic lineages of *M. tuberculosis* clinical strains should be performed to further investigate polymorphisms in the proteins that compose these vaccine candidates and examine whether this diversity creates variation in regions of the proteins predicted to serve as epitopes.

To summarize, our study found notable differences in the predicted coverage of Ag85B-ESAT-6, Ag85B-TB10.4, and Mtb72f, with Ag85B-TB10.4 predicted to have the best overall population coverage. The findings of this study highlight bioinformatics as a useful approach to evaluating vaccine candidates before they reach the costly stages of clinical trials. Although epitope binding prediction programs are imperfect, they offer a low-cost and low-risk approach to exploring and comparing vaccine coverage, and may offer important insights into the pre-clinical stages of vaccine development and testing. For example, our analysis of the population coverage of the three vaccine candidates identified several populations where 30% or more of the population is expected to have two alleles of concern at the same HLA locus, demonstrating that there are populations where the variation in the host’s ability to present vaccine epitopes may have an important impact on vaccine efficacy. Such information may guide decisions on which populations to focus on during clinical trials. Future studies should, therefore, incorporate host and pathogen diversity into the creation of epitope-driven vaccines as well as into testing of their global coverage.

## Materials and Methods

### Selecting Programs for Class I and Class II Epitope Binding Prediction

This study took a consensus approach to epitope binding prediction, which incorporates several algorithms to generate more accurate binding predictions than single-method approaches [32]. Class I epitope binding predictions were generated with NetMHCcons, a server that incorporates artificial neural network-based (ANN), pan-specific ANN, and matrix-based methods to give highly accurate predictions [26], and that was recently determined to be the best available method for generating MHC Class I predictions [27]. Class II epitope binding predictions were generated with a set of eight programs: ARB, NetMHCII, NetMHCIIpan, ProPred, SVRMHCII, MHCPred, RankPEP, and Vaxign [13]. The methods of these programs include artificial neural networks [33], support vector machine regression models [34], [35], matrix-based models [36], and partial least squares models [37], [38].

For Class I predictions, a binding cutoff of IC_50_≤500 was used [26]. For Class II predictions, default binding cutoffs were used for programs that predicted binding in a yes/no fashion. For programs that generated IC_50_ or pIC_50_ values for binding predictions, IC_50_≤500 was used as the binding cutoff [39]. The only program that did not fall into either of the preceding categories was ProPred, for which the recommended 3% best scoring peptides among all possible epitopes was used as the cutoff. Class II binding predictions used the same binding cutoffs used in [13].

### Selecting High-frequency Alleles

This study tested 89 HLA-A, –B, and –C alleles and 34 HLA-DRB1 alleles [13], representing the three most prevalent HLA–A, HLA–B, HLA–C, and HLA-DRB1 alleles in each population in the Allele*Frequencies in WorldWide Populations database (www.allelefrequencies.net) from the WHO 22 countries of high TB burden (Tables S1, S2, S3, S4) [24], [25].

### Supertype Alleles

Nine Class I HLA supertype alleles (A*0101, A*0201, A*0301, A*2601, B*0702, B*1501, B*2705, B*4001, and B*5801) and eight HLA-DRB1 supertype alleles (DRB1*0101, *0301, *0401, *0701, *0801, *1101, *1301, and *1501) were used in the comparative analysis of Ag85B-ESAT-6, Ag85B-TB10.4, and Mtb72f. These supertype alleles represent the primary functional binding motifs of most Class I alleles and nearly all HLA-DRB1 alleles [28], [29].

### Epitope Binding Predictions

Class I and II epitope binding predictions for vaccine candidates were obtained by entering all conserved *M. tuberculosis* epitopes from Ag85B-ESAT-6, Ag85B-TB10.4, and Mtb72f into the most recently updated versions of one Class I and eight Class II programs. Protein sequences for Ag85B-ESAT-6 and Ag85B-TB10.4 were derived from the H37Rv reference strain, as a previous study of 91 clinical strains–defined by IS*6110* restriction fragment length polymorphism analysis and spoligotyping–found no phenotypic diversity in the three component proteins of Ag85B-ESAT-6 and Ag85B-TB10.4 [11]. The conserved epitopes for Mtb72f were derived from two conserved segments of the pepA protein and the complete list of conserved PPE18 epitopes as reported in [13]. All Class I binding predictions were generated by NetMHCcons, while Class II binding predictions came from different subsets of the eight programs for each allele because not all programs predicted binding for all 34 DRB1 alleles.

Since our publication of Mtb72f epitope binding predictions in [13], five of the eight epitope binding prediction programs used in this study (ARB, NetMHCII, NetMHCIIpan, MHCPred, and RankPEP) were updated. To permit the comparison of prediction results among Ag85B-ESAT-6, Ag85B-TB10.4, and Mtb72f, new epitope binding predictions were completed for the conserved regions of Mtb72f, as defined in [13]. Program updates did not change the conclusions of [13], although minor changes were observed in the predicted bindings per allele.

The predictions generated by each program were compiled in Excel 2007 (Microsoft, Redmond, WA). If binding prediction programs predicted multiple epitopes of differing length but with the same nonamer binding core, the minimum core required to bind class II HLA, unique nonamer cores were counted only once to avoid overestimation of bound epitopes per allele. We screened epitope binding prediction results for HLA alleles of concern, defined by McNamara and colleagues as variants predicted to bind four or fewer conserved vaccine epitopes, and compared the results for the three vaccine candidates.

### Assessment of Population Coverage

The allele frequencies of all HLA–A, B, C, and DRB1 alleles were considered to determine the expected coverage of Ag85B-ESAT-6, Ag85B-TB10.4, and Mtb72f in populations of high TB burden. All populations were classified as being of lesser, moderate, or great concern for reduced vaccine coverage. Populations of moderate concern were defined as populations where the frequency of individuals with two HLA alleles of the same HLA gene that are both alleles of concern–alleles predicted to bind four or fewer vaccine epitopes–was 10% or greater and less than 30%. Populations of great concern were defined as those where the frequency of having both HLA alleles be alleles of concern was 30% or greater. All remaining populations were classified as being of populations of lesser concern. Phenotypic frequencies were calculated using allele frequencies from the Allele*frequencies database under the assumption of Hardy-Weinberg equilibrium.

### Control Proteins

To test that observed variations in predicted epitope bindings were a function of the vaccine proteins rather than an artifact of the prediction programs, we generated Class I and II epitope binding predictions for three non-mycobacterium control proteins. The control proteins were of similar amino acid length to the three vaccine candidates, and included: 1) Dihydrolipoyllysine-residue succinyltransferase (389 aa) of *Neisseria meningitides,* 2) Cytochrome B (380 aa) of *Homo sapiens*, and 3) TPA_exp: BimA (373 aa) of *Burkholderia mallei* (www.ncbi.nlm.nih.gov). Two-way ANOVA was performed on control and test protein epitope predictions for all Class I and Class II alleles analyzed to assess the sources of variation in the number of epitopes from each protein predicted to bind to each HLA allele.

## Supporting Information

Table S1 Epitope binding predictions of Ag85B-ESAT-6, Ag85B-TB10.4, and Mtb72f vaccines and control proteins TPA_exp: BimA, Succinyltransferase, and Cytochrome B to high-frequency HLA-A alleles among TB high-burden populations.
(DOCX)

Table S2 Epitope binding predictions of Ag85B-ESAT-6, Ag85B-TB10.4, and Mtb72f vaccines and control proteins TPA_exp: BimA, Succinyltransferase, and Cytochrome B to high-frequency HLA-B alleles among TB high-burden populations.
(DOCX)

Table S3 Epitope binding predictions of Ag85B-ESAT-6, Ag85B-TB10.4, and Mtb72f vaccines and control proteins TPA_exp: BimA, Succinyltransferase, and Cytochrome B to high-frequency HLA-C alleles among TB high-burden populations.
(DOCX)

Table S4 Epitope binding predictions for Ag85B-ESAT-6, Ag85B-TB10.4, and Mtb72f vaccines and control proteins TPA_exp: BimA, Succinyltransferase, and Cytochrome B to high-frequency HLA-DRB1 alleles among TB high-burden populations.
(DOCX)
