# Correction: Comparison of the Predicted Population Coverage of Tuberculosis Vaccine Candidates Ag85B-ESAT-6, Ag85B-TB10.4, and Mtb72f via a Bioinformatics Approach

**DOI:** 10.1371/annotation/ff089043-990a-48c2-a90f-15606c11cc98

**Published:** 2012-10-08

**Authors:** Jose Davila, Lucy A. McNamara, Zhenhua Yang

We found that for Class I epitope prediction data, different HLA–A, −B, and −C alleles account for 51.59% of the variation in epitopes predicted to bind (F = 10.55, p<0.0001) while the specific vaccine or control protein analyzed accounts for 23.52% of the variation (F = 84.16, p<0.0001). For the Class II predictions, different HLA-DRB1 alleles account for 66.55% of the variation in epitopes predicted to bind (F = 37.29, p<0.0001) while the specific vaccine or control protein analyzed accounts for 24.52% of the variation (F = 90.70, p<0.0001).

