# Supplementary material for: Comparison of the Predicted Population Coverage of Tuberculosis Vaccine Candidates Ag85B-ESAT-6, Ag85B-TB10.4, and Mtb72f via a Bioinformatics Approach
Source: PLoS One. 2012 Jul 17;7(7):e40882. doi: 10.1371/journal.pone.0040882 (PMC3398899; doi:10.1371/journal.pone.0040882)
Supplement: Table S1 — Epitope binding predictions of Ag85B-ESAT-6, Ag85B-TB10.4, and Mtb72f vaccines and control proteins TPA_exp: BimA, Succinyltransferase, and Cytochrome B to high-frequency HLA-A alleles among TB high-burden populations. (DOCX) [file pone.0040882.s001.docx]

Table S1: Epitope binding predictions of Ag85B-ESAT-6, Ag85B-TB10.4, and Mtb72f vaccines and control proteins TPA_exp: BimA, Succinyltransferase, and Cytochrome B to high-frequency HLA-A alleles among TB high-burden populations.

| **HLA-A allele** | **Ag85B-ESAT-6** | **Ag85B-TB10.4** | **Mtb72f** | **TPA_exp: BimA** | **Succinyl- transferase** | **Cyto-chrome B** | **Population** |
| --- | --- | --- | --- | --- | --- | --- | --- |
| A*0101 | 6 | 6 | 9 | 4 | 5 | 9 | Brazil; Russia; India; Pakistan Pathan and Sindhi; Kenya Nandi, South Africa Natal Tamil; Uganda Kampala |
| A*0201 | 9 | 10 | 5 | 5 | 6 | 38 | Brazil; China; India; Russia; Thailand; South Africa Tswana; Vietnam Hanoi; Kenya; Uganda Kampala; Pakistan Brahui and Kalash; Philippines Ivatan |
| A*0203 | 18 | 16 | 20 | 16 | 24 | 43 | China Guangxi, Yunnan, Southwest Dai; Thailand |
| A*0206 | 15 | 19 | 25 | 8 | 21 | 55 | Pakistan Kalash |
| A*0207 | 4 | 7 | 4 | 2 | 3 | 20 | China Guizhou, Southwest Dai, Yunnan, and Guangxi; Thailand |
| A*0222 | 25 | 26 | 24 | 18 | 31 | 70 | India West Bhil |
| A*0301 | 1 | 1 | 1 | 6 | 10 | 10 | Brazil Minas Gerais and Parana; Russia; India Jalpaiguri Toto, Kerala, Tamil Nadu Nadar, and New Delhi; Pakistan Burusho |
| A*1101 | 3 | 3 | 1 | 5 | 10 | 10 | Bangladesh Dhaka Bangalee; Brazil Parana Oriental and Sao Paulo; China; India; Indonesia Sundanese and Javanese; Pakistan; South Africa Natal Tamil; Thailand; Vietnam Hanoi |
| A*2301 | 4 | 6 | 1 | 2 | 3 | 29 | Kenya Luo; South Africa Natal Zulu; Zimbabwe Harare Shona; Uganda Kampala |
| A*2402 | 6 | 7 | 2 | 1 | 1 | 26 | Bangladesh Dhaka Bangalee; Brazil; China; India; Indonesia Java; Pakistan Burusho, Pathan, and Sindhi; Philippines Ivatan; Russia; South Africa Natal Tamil; Thailand Northeast; Vietnam Hanoi |
| A*2407 | 6 | 7 | 1 | 2 | 2 | 28 | Indonesia Sundanese and Javanese; India West Bhil; China Yunnan |
| A*2420 | 7 | 8 | 1 | 1 | 2 | 27 | China Guangdong Province Meizhou Han |
| A*2601 | 8 | 10 | 5 | 6 | 8 | 15 | India Jalpaiguri Toto; Pakistan Baloch |
| A*2902 | 8 | 8 | 4 | 5 | 4 | 24 | South Africa Tswana and Natal Zulu |
| A*3001 | 7 | 10 | 8 | 13 | 19 | 14 | Brazil Parana; China Shandong; India West Coast Parsi; Kenya; Pakistan Kalash and Karachi Parsi; Russia Sakhalin Island Nivkhi; South Africa Tswana |
| A*3002 | 12 | 13 | 3 | 6 | 4 | 13 | Zimbabwe Harare Shona |
| A*3101 | 1 | 1 | 1 | 10 | 7 | 7 | Brazil Terena; India Andhra Pradesh Golla and Tamil Nadu Nadar; Russa Tuva |
| A*3108 | 9 | 10 | 2 | 7 | 4 | 40 | India West Coast Parsi |
| A*3201 | 6 | 4 | 10 | 10 | 12 | 31 | Pakistan Brahui |
| A*3301 | 1 | 1 | 0 | 5 | 4 | 5 | Bangladesh Dhaka Bangalee; China Harbin Korean; India Kerala; Pakistan Karachi Parsi |
| A*3303 | 2 | 2 | 1 | 6 | 7 | 5 | China South Han and Yunnan Province; India Mumbai Maratha, West Bhil, West Coast Parsi; Indonesia Sundanese and Javanese; Pakistan Baloch and Bursho; Vietnam Hanoi |
| A*3401 | 12 | 13 | 12 | 13 | 14 | 22 | Philippines Ivatan |
| A*6801 | 8 | 6 | 1 | 13 | 15 | 9 | Brazil Pernambuco and Terena; India Delhi |
| A*6802 | 10 | 14 | 22 | 15 | 20 | 24 | Kenya; South Africa Natal Zulu; Zimbabwe Harare Shona |
| A*7401 | 1 | 1 | 0 | 7 | 8 | 7 | Kenya; Uganda Kampala |
