# Supplementary material for: Comparison of the Predicted Population Coverage of Tuberculosis Vaccine Candidates Ag85B-ESAT-6, Ag85B-TB10.4, and Mtb72f via a Bioinformatics Approach
Source: PLoS One. 2012 Jul 17;7(7):e40882. doi: 10.1371/journal.pone.0040882 (PMC3398899; doi:10.1371/journal.pone.0040882)
Supplement: Table S2 — Epitope binding predictions of Ag85B-ESAT-6, Ag85B-TB10.4, and Mtb72f vaccines and control proteins TPA_exp: BimA, Succinyltransferase, and Cytochrome B to high-frequency HLA-B alleles among TB high-burden populations. (DOCX) [file pone.0040882.s002.docx]

Table S2: Epitope binding predictions of Ag85B-ESAT-6, Ag85B-TB10.4, and Mtb72f vaccines and control proteins TPA_exp: BimA, Succinyltransferase, and Cytochrome B to high-frequency HLA-B alleles among TB high-burden populations.

| **HLA-B allele** | **Ag85B-ESAT-6** | **Ag85B-TB10.4** | **Mtb72f** | **TPA_exp: BimA** | **Succinyl- transferase** | **Cyto-chrome B** | **Population** |
| --- | --- | --- | --- | --- | --- | --- | --- |
| B*0702 | 9 | 11 | 9 | 5 | 12 | 10 | Brazil Belo Horizonte Caucasian, Parana, Pernambuco; India Jalpaiguri Toto, Kerala; Russia; Uganda Kampala; Vietnam Hanoi |
| B*0705 | 7 | 10 | 13 | 5 | 12 | 9 | India Mumbai Maratha; Vietnam Hanoi Kinh |
| B*0801 | 1 | 2 | 2 | 6 | 6 | 14 | Brazil Belo Horizonte Caucasian; India Jalpaiguri Toto and Kerala Kurchiya; Pakista Burusho, Pathan, Sindhi; Uganda Kampala |
| B*1301 | 7 | 10 | 1 | 1 | 7 | 14 | China; Pakistan Karachi Parsi; Russia South Ural; Thailand Northeast |
| B*1302 | 6 | 7 | 2 | 3 | 4 | 19 | China Beijing; India West Coast Parsi |
| B*1402 | 13 | 15 | 5 | 7 | 4 | 18 | India Jalpaiguri Toto; Pakistan Karachi Parsi |
| B*1405 | 7 | 11 | 6 | 5 | 5 | 14 | India West Coast Parsi |
| B*1501 | 11 | 18 | 7 | 7 | 5 | 22 | Bangladesh Dhaka Bangalee; Brazil; China; India Kerala and Tamil Nadu Nadar; Philippines Ivatan; Russia Arkhangelsk Pomor and Murmansk Saomi; South Arica Tswana; Tanzania Dodoma Kongwa; Thailand Northeast; Vietnam Hanoi |
| B*1502 | 14 | 15 | 7 | 5 | 4 | 13 | China; Indonesia Sundanese and Javanese; Philippines Ivatan; Thailand; Vietnam Hanoi |
| B*1503 | 43 | 52 | 28 | 31 | 29 | 75 | Kenya Luo; South Africa Natal Zulu; Uganda Kampala |
| B*1507 | 9 | 13 | 6 | 5 | 6 | 20 | China Yunnan Province |
| B*1510 | 11 | 15 | 10 | 8 | 3 | 14 | Zimbabwe Harare Shona |
| B*1511 | 11 | 13 | 9 | 7 | 6 | 15 | China Beijing |
| B*1513 | 12 | 15 | 9 | 7 | 2 | 26 | Indonesia Sundanese and Javanese |
| B*1521 | 11 | 16 | 8 | 9 | 5 | 17 | Indonesia Java |
| B*1532 | 10 | 14 | 8 | 5 | 4 | 21 | China Yunnan Province Wa |
| B*1801 | 7 | 10 | 1 | 1 | 7 | 4 | Brazil |
| B*2704 | 3 | 5 | 1 | 7 | 5 | 6 | China Tibet Region; Russia Chuvash, Sakhalin Island Nivkhi; Pakistan Kalash |
| B*2705 | 1 | 2 | 1 | 5 | 4 | 5 | Supertype* |
| B*3501 | 13 | 16 | 12 | 9 | 7 | 19 | Bangladesh Dhaka Bangalee; Brazil; China Qinghai and Yunnan Provinces; India; Pakistan; Russia |
| B*3503 | 14 | 18 | 11 | 7 | 15 | 17 | India Delhi, North, and West Bhil |
| B*3505 | 13 | 16 | 11 | 9 | 8 | 21 | China Yunnan Province; Indonesia Sundanese and Javanese |
| B*3506 | 10 | 14 | 11 | 6 | 14 | 16 | Brazil Terena |
| B*3901 | 8 | 10 | 9 | 9 | 5 | 12 | Supertype* |
| B*3903 | 11 | 13 | 10 | 6 | 5 | 12 | Brazil Terena |
| B*3906 | 7 | 9 | 11 | 6 | 4 | 16 | Russia Bering Island Aleut |
| B*4001 | 6 | 8 | 2 | 1 | 14 | 5 | China; India; Pakistan Baloch, Brahui, Sindhi; Philippines Ivatan; Russia Sakhalin Island Nivkhi; Thailand |
| B*4002 | 6 | 10 | 0 | 1 | 9 | 6 | Russia Bering Island Aleut and Tuva; China Inner Mongolian |
| B*4006 | 5 | 10 | 0 | 1 | 12 | 6 | India; South Africa Natal Tamil |
| B*4201 | 8 | 10 | 12 | 5 | 10 | 14 | South Africa Natal Zulu; Kenya |
| B*4403 | 7 | 9 | 1 | 1 | 9 | 3 | Brazil; Russia Moscow, South Ural, Northwest; Bangladesh Dhaka Bangalee; China Yunnan Province Han; South Africa Tswana, Natal Zulu; Thailand; India New Delhi; Indonesia Java |
| B*4404 | 8 | 11 | 1 | 1 | 11 | 5 | India West Coast Parsi |
| B*4406 | 16 | 17 | 11 | 7 | 4 | 20 | India Mumbai Maratha |
| B*4501 | 4 | 5 | 2 | 4 | 8 | 3 | Tanzania Dodoma Kongwa; Uganda Kampala; Zimbabwe Harare Shona |
| B*4601 | 12 | 15 | 13 | 6 | 6 | 15 | China; Thailand; Vietnam Hanoi |
| B*4801 | 8 | 12 | 3 | 4 | 3 | 16 | China North Han; Russia Nenet and Sakhalin Island Nivkhi |
| B*4803 | 6 | 9 | 6 | 2 | 5 | 12 | Brazil Terena |
| B*5101 | 8 | 9 | 11 | 3 | 11 | 25 | Brazil; China; India; Pakistan; Russia South Ural Bashkir; South Africa Natal Tamil |
| B*5201 | 3 | 4 | 6 | 5 | 5 | 27 | India Andhra Pradesh, Kerala, North; China Yunnan Province |
| B*5301 | 9 | 10 | 7 | 8 | 3 | 20 | Kenya; Uganda Kampala; Zimbabwe Harare Shona |
| B*5303 | 10 | 13 | 10 | 8 | 6 | 22 | India West Bhil |
| B*5502 | 7 | 12 | 18 | 7 | 15 | 18 | China Guizhou Province Miao |
| B*5601 | 7 | 12 | 18 | 8 | 15 | 18 | China Yunnan Province Lisu |
| B*5701 | 8 | 9 | 4 | 6 | 6 | 23 | India Kerala Malapandaram; South Africa Natal Tamil |
| B*5801 | 11 | 11 | 4 | 8 | 3 | 20 | China; India Delhi, Kerala, Khandesh Region, West Bhil; Kenya; Pakistan Baloch; Russia Tuva; South Africa Tswana; Tanzania Dodoma Kongwa |
| B*5802 | 8 | 9 | 3 | 8 | 4 | 22 | Kenya Luo and Nandi; Uganda Kampala |

*HLA-B*2705 and B*3901 are supertype alleles that were not among the top three most common HLA Class I alleles in any populations from the 22 countries of high TB burden.
