# Supplementary material for: Comparison of the Predicted Population Coverage of Tuberculosis Vaccine Candidates Ag85B-ESAT-6, Ag85B-TB10.4, and Mtb72f via a Bioinformatics Approach
Source: PLoS One. 2012 Jul 17;7(7):e40882. doi: 10.1371/journal.pone.0040882 (PMC3398899; doi:10.1371/journal.pone.0040882)
Supplement: Table S3 — Epitope binding predictions of Ag85B-ESAT-6, Ag85B-TB10.4, and Mtb72f vaccines and control proteins TPA_exp: BimA, Succinyltransferase, and Cytochrome B to high-frequency HLA-C alleles among TB high-burden populations. (DOCX) [file pone.0040882.s003.docx]

Table S3: Epitope binding predictions of Ag85B-ESAT-6, Ag85B-TB10.4, and Mtb72f vaccines and control proteins TPA_exp: BimA, Succinyltransferase, and Cytochrome B to high-frequency HLA-C alleles among TB high-burden populations.

| **HLA-C allele** | **Ag85B-ESAT-6** | **Ag85B-TB10.4** | **Mtb72f** | **TPA_exp: BimA** | **Succinyl- transferase** | **Cyto-chrome B** | **Population** |
| --- | --- | --- | --- | --- | --- | --- | --- |
| C*0102 | 6 | 9 | 9 | 7 | 4 | 19 | China; India Kerala Hindu Pulaya; Thailand; Vietnam Hanoi |
| C*0202 | 7 | 8 | 8 | 7 | 6 | 18 | India Tamil Nadu Nadar |
| C*0302 | 34 | 44 | 43 | 29 | 35 | 66 | China Shandong Province; India; Pakistan Brahui; Vietnam Hanoi |
| C*0303 | 10 | 10 | 13 | 12 | 11 | 17 | China Inner Mongolian Region; Russia Murmansk Saomi and Sakhalin Island Nivkhi |
| C*0304 | 10 | 10 | 13 | 12 | 11 | 17 | Brazil Terena; China; Russia |
| C*0401 | 6 | 5 | 4 | 1 | 7 | 12 | Brazil Pernambuco and Terena; China; India; Kenya; Pakistan; Philippines Ivatan; Russia; Thailand Northeast; Uganda Kampala; Zimbabwe Harare |
| C*0501 | 7 | 6 | 8 | 2 | 10 | 10 | India Khandesh Region Pawra |
| C*0602 | 7 | 8 | 3 | 3 | 4 | 19 | Brazil Pernambuco; China; India; Kenya; Pakistan Kalash, Karachi, Pathan; Russia Arkhangelsk Pomor, Moscow, Tuva; South Africa Natal Tamil; Uganda Kampala; Zimbabwe Harare Shona |
| C*0701 | 9 | 10 | 7 | 9 | 4 | 22 | Brazil Pernambuco; India; Kenya; Pakistan; Russia Moscow; South Africa Natal Tamil; Thailand Northeast; Uganda Kampala; Vietnam Hanoi; Zimbabwe Harare Shona |
| C*0702 | 10 | 12 | 4 | 6 | 6 | 20 | Brazil Terena; China; India Delhi and Tamil Nadu Nadar; Pakistan Brahui and Burusho; Philippines Ivatan; Russia; Thailand; Vietnam Hanoi Kinh |
| C*0704 | 9 | 10 | 3 | 5 | 3 | 17 | India Mumbai Maratha |
| C*0717 | 9 | 11 | 3 | 4 | 6 | 21 | China Guangdong Province Meizhou Han |
| C*0801 | 9 | 8 | 7 | 3 | 8 | 17 | China; Philippines Ivatan; Russia Nenet and Sakhalin Island Nivkhi; Thailand; Vietnam Hanoi |
| C*0802 | 8 | 7 | 8 | 6 | 10 | 12 | Pakistan Karachi Parsi |
| C*1202 | 19 | 25 | 25 | 18 | 13 | 37 | China Shandong Province; India; Pakistan Sindhi; Russia Moscow |
| C*1203 | 13 | 16 | 8 | 10 | 8 | 26 | Pakistan Burusho |
| C*1502 | 8 | 10 | 12 | 3 | 6 | 13 | South Africa Natal Tamil |
| C*1507 | 8 | 9 | 15 | 8 | 10 | 16 | India New Delhi |
