# Supplementary material for: Comparison of the Predicted Population Coverage of Tuberculosis Vaccine Candidates Ag85B-ESAT-6, Ag85B-TB10.4, and Mtb72f via a Bioinformatics Approach
Source: PLoS One. 2012 Jul 17;7(7):e40882. doi: 10.1371/journal.pone.0040882 (PMC3398899; doi:10.1371/journal.pone.0040882)
Supplement: Table S4 — Epitope binding predictions for Ag85B-ESAT-6, Ag85B-TB10.4, and Mtb72f vaccines and control proteins TPA_exp: BimA, Succinyltransferase, and Cytochrome B to high-frequency HLA-DRB1 alleles among TB high-burden populations. (DOCX) [file pone.0040882.s004.docx]

Table S4: Epitope binding predictions for Ag85B-ESAT-6, Ag85B-TB10.4, and Mtb72f vaccines and control proteins TPA_exp: BimA, Succinyltransferase , and Cytochrome B to high-frequency HLA-DRB1 alleles among TB high-burden populations.

| **DRB1 Allele** | **Ag85B-ESAT-6** | **Ag85B-TB10.4** | **Mtb72f** | **TPA_exp: BimA** | **Succinyl- transferase** | **Cyto-chrome B** | **Population** |
| --- | --- | --- | --- | --- | --- | --- | --- |
| *0101 | 68 | 68.5 | 66.5 | 46.5 | 68 | 92.5 | Russian Chuvash, Aleuts and Tuva; Indian Islamic populations |
| *0102 | 8 | 11 | 20 | 5 | 12 | 33 | Ethiopia (Amhara) |
| *0301 | 7.5 | 9.5 | 9.5 | 9.5 | 20 | 15.5 | Brazil; northern China; Ethiopia (Oromo); northern and eastern India; northwestern Russia; Thailand (Bangkok); South Africa (Venda) |
| *0302 | 6 | 9 | 0 | 5 | 8 | 12 | South Africa (Venda) |
| *0401 | 29.5 | 32 | 22.5 | 20 | 23.5 | 48 | Chinese Inner Mongolian Evenki; Russian Chukchi, Eskimo, Koryak, Buryat, and Negidal populations |
| *0403 | 6 | 10 | 2 | 4 | 5 | 23 | Russian Buryat and Nganasan populations |
| *0404 | 14 | 13 | 15 | 10 | 14 | 59 | Brazil Kaingang indigenous population |
| *0405 | 23.5 | 23 | 11.5 | 8 | 16 | 50 | Philippines |
| *0411 | 14 | 17 | 4 | 7 | 15 | 48 | Brazil indigenous populations |
| *0701 | 23 | 20 | 13 | 22 | 17 | 64 | Brazil; Northern and Eastern China; Democratic Republic of the Congo; Ethiopia; Indonesia (Java); Russian Bearian Island Aleuts, Chuvash, Siberia; Thailand (Bangkok); Vietnam (Hanoi) |
| *0801 | 3 | 6 | 10 | 8 | 10 | 17 | Russia (Kets) |
| *0802 | 8.5 | 10.5 | 8.5 | 14.5 | 14.5 | 20 | Brazil indigenous populations; Russian Eskimos |
| *0803 | 43 | 43 | 37 | 39 | 58 | 77 | China (Yunnan Province) |
| *0804 | 20 | 20.5 | 24 | 21.5 | 33 | 40.5 | Brazil East Amazon indigenous populations |
| *0807 | 3 | 5 | 4 | 6 | 12 | 16 | Brazil Guarani Kaiowa and Ticuna indigenous populations |
| *0901 | 28 | 28.5 | 26.5 | 22.5 | 23.5 | 40.5 | Brazil Southeast Caucasian population; China; Russia (Siberia); Thailand; Vietnam (Hanoi) |
| *1001 | 45 | 51 | 37 | 32 | 37 | 62 | Northern and eastern India; Russian Buryat population |
| *1101 | 14 | 16 | 8 | 9 | 11 | 44 | Northeast Brazil; Northern and central China; Democratic Republic of the Congo; northern India; Indonesia (Molucca and Nusa Tenggara); Russian Evenks, Nganasan, and Tuva populations; Zimbabwe (Harare) |
| *1201 | 32 | 34 | 29 | 30 | 47 | 64 | China (Southern and Harbin); northeast India; Russia (Siberia) |
| *1202 | 37 | 39 | 36 | 35 | 42 | 91 | Southern and central China; Hong Kong and Singapore; Indonesia; Philippines; Thailand, Vietnam (Hanoi) |
| *1301 | 8 | 8.5 | 12 | 8 | 10.5 | 15 | Brazil; China (Xinjiang); Democratic Republic of the Congo; India (Andhra Pradesh); Russia (Kets, Khanty-Mansi); South Africa (Venda); Zimbabwe (Harare) |
| *1302 | 12.5 | 9 | 14.5 | 11 | 14 | 21 | Brazil Southeast Mulattos; Democratic Republic of the Congo; Ethiopia |
| *1303 | 83 | 82 | 79 | 76 | 89 | 112 | Democratic Republic of the Congo; Northeast India |
| *1401 | 6 | 8 | 4 | 8 | 10 | 33 | Southern China; Northern India; Philippines; Russian Nivkhi and Evenki, Udege, and Ulchi; Vietnamese Muong |
| *1402 | 27 | 28 | 19 | 21 | 29 | 54 | Brazil Xavantes, Guarani, and Terena; Russian Chukchi, Eskimos, Koryaks, Nivkhi, and Udege |
| *1403 | 10 | 11 | 0 | 9 | 11 | 25 | Chinese Drung; Russian Evenki and Kets |
| *1404 | 17 | 15 | 7 | 15 | 22 | 54 | China Naxi and Lisu; India (Delhi) |
| *1405 | 22 | 23 | 22 | 17 | 27 | 56 | Chinese Wa population |
| *1413 | 64 | 68 | 49 | 55 | 80 | 97 | Brazil Guarani M bya population |
| *1501 | 14 | 14 | 10 | 11 | 18 | 63 | China; Hong Kong and Singapore; India; Indonesia; Russia (Siberia); Northeast Thailand |
| *1502 | 4.5 | 7 | 3 | 4 | 4 | 27 | Chinese Jino population; India; Indonesia; Philippines; Thailand; Vietnam |
| *1503 | 17 | 20 | 12 | 14 | 21 | 69 | Zimbabwe (Harare) |
| *1504 | 16 | 19 | 7 | 14 | 21 | 68 | Chinese Nu and Va populations |
| *1602 | 32 | 18.5 | 14 | 20 | 24 | 79 | Brazil Xavantes, Guarani, Kaingang, Terena, and Ticuna; Chinese Maonan and Miao; Northern Thailand; Vietnamese Muong |

Median numbers of predicted bindings per allele are shown.
